# Sharing big biomedical data

**DOI:** 10.1186/s40537-015-0016-1

**Published:** 2015-06-27

**Authors:** Arthur W Toga, Ivo D Dinov

**Affiliations:** Laboratory of Neuro Imaging, Institute of Neuroimaging and Informatics, Keck School of Medicine of USC, University of Sothern California, 2001 North Soto Street-Room 102, Los Angeles, CA 90033 USA; Statistics Online Computaitonal Resource, University of Michigan, UMSN, 400 North Ingalls, Room 4341, Ann Arbor, 48109-5482 MI USA

**Keywords:** Big data, Policy, Sharing, Analytics, Privacy

## Abstract

**Background:**

The promise of Big Biomedical Data may be offset by the enormous challenges in handling, analyzing, and sharing it. In this paper, we provide a framework for developing practical and reasonable data sharing policies that incorporate the sociological, financial, technical and scientific requirements of a sustainable Big Data dependent scientific community.

**Findings:**

Many biomedical and healthcare studies may be significantly impacted by using large, heterogeneous and incongruent datasets; however there are significant technical, social, regulatory, and institutional barriers that need to be overcome to ensure the power of Big Data overcomes these detrimental factors.

**Conclusions:**

Pragmatic policies that demand extensive sharing of data, promotion of data fusion, provenance, interoperability and balance security and protection of personal information are critical for the long term impact of translational Big Data analytics.

## Introduction

Large-scale, data-intensive research enterprises in the health sciences such as the Encyclopedia of DNA Elements (ENCODE) [1], Model Organism Protein Expression Database (MOPED) [2], Alzheimer’s Disease Neuroimaging Initiative (http://adni.loni.usc.edu/) [3], Early Detection Research Network (EDRN) [4], Parkinson’s Progression Markers Initiative (PPMI) [5], database of Genotypes and Phenotypes (dbGaP) [6], and ClinicalTrials.gov [7, 8] exemplify several models that have vastly improved data management, data sharing and distributed access of imaging, biological, genetics and clinical data on a broad array of human diseases [2, 9–17]. The resulting increase in utilization has been driven largely by transition to high information density [18]; the demand for multi-scale, multi-modal, large N data in the investigation of fundamental disease processes [19]; the necessity of applying methodologies and insights from multiple disciplines in order to adequately integrate, query, analyze and interpret the data [14]; and the movement of science in general toward freely and openly available information [20]. By now, the electronic collection, organization, annotation, storage, and distribution of heterogeneous data are essential activities in the contemporary biomedical, clinical, and translational discovery processes.

Big Data stresses an already challenging set of requirements for data sharing. In the biosciences, Big Data refers to large-scale data sets with complex-organization that arise from different sources in many fields (e.g., genomics, physiology, imaging, health informatics). The core features of Big Data include data-size, data incompleteness, data incompatibility, data heterogeneity and incongruent sampling. Big Data sharing requires innovative policies and clear guidelines that promote cooperation and trans-disciplinary interactions in spite of the technical, financial, security and other complexities introduced by Big Data.

### How big is Big Data?

**Fig. 1:**
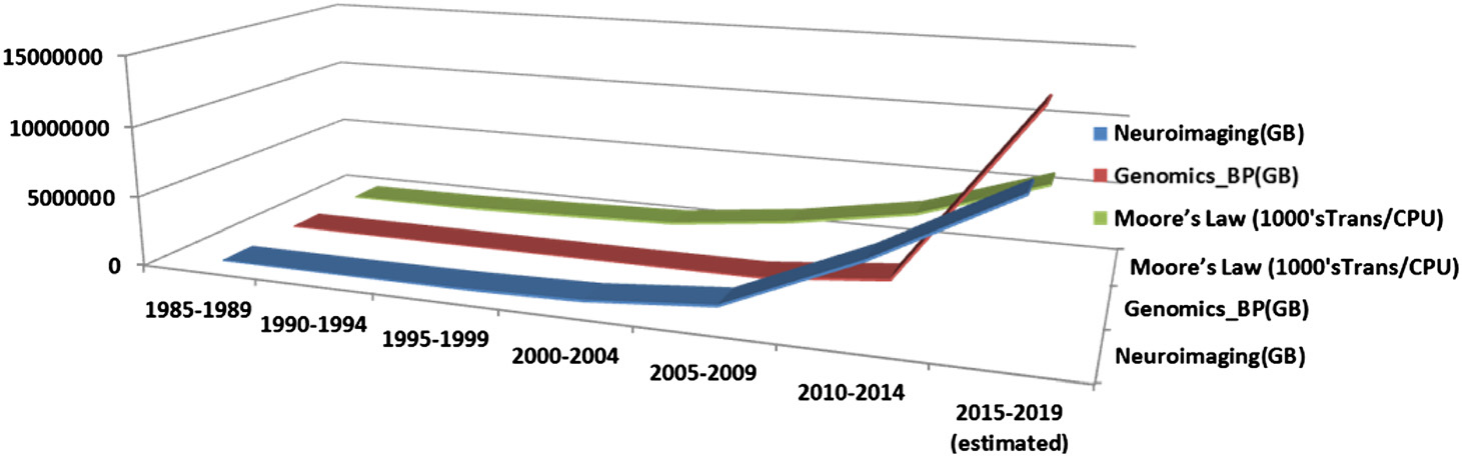
(Kryder’s law) Exponential growth of neuroimaging and genomics data, relative to increase of number of transistors per chip (Moore’s law) [21]. The misalignment between rate of increase of computational power and volume of data is the result of rapid technological advances improvements in data resolution, streaming efficiency and censoring equipment. By 2015 more than a 106 whole human genomes will be sequenced totaling over 100 PB and many neuroimaging studies will generate over 1 TB of data daily

Even data from a single individual may be unwieldy with certain high-data-density methods (e.g., whole genome sequencing) producing Big Data, or by the ever-increasing temporal or spatial resolution (e.g., as in magnetic resonance imaging) of acquisition devices. The expanding volume, complexity, and derivatives (a measure of the generated derived data) of Big Data present scale-intensified versions of familiar as well as newly emerging challenges for data sharing. Figure 1 shows the exponential growth (Kryder’s law, which significantly outpaces the expected increase of computational power, Moore’s law) [21] for neuroimaging and genomics data.

In addition, ultra-large data sets can be unit-wise manageable, but when hundreds or thousands of subjects are combined during (meta)analysis, the raw and derived data size and complexity may exceed or stress extant resources. This article surveys an illuminating sample of those challenges, along with many of the considerations necessary to create a fair, equitable, responsible and practical set of policies to serve the individual investigator, the research project, the funder and the greater scientific community. In some cases policies can easily be viewed as detrimental to the individual but advantageous to the group, or vice versa. How should a policy prioritize Big Data requests that by their very nature reduce access by others? Even technical implementations or financial limitations can have an adverse effect, such as whether the computational infrastructure at a research facility or consortium to collect, manage, and disseminate data can overcome service bottlenecks (bandwidth and latency) when hundreds of investigators request terabytes and, prospectively, petabytes of data at the same time. Or whether only relatively wealthy investigator groups have access to the hardware needed to access, copy, process or analyze shared Big Data.

Existing policies on data sharing are often merely statements of the goal - ‘We should share data.’ Without intending to be critical, many funders simply stipulate sharing as a requirement. And the sharing plan often included in grant proposals is typically simplistic, usually under- or even un-funded and rarely considers all of the issues required to fully or fairly share data (or for that matter protocols, results and computational infrastructure). Funding for sustainable data stewardship is a major issue (and more so with Big Data) as federal and foundation support is inadequate [22]. Some applicants merely describe a plan to deposit the data in some web-based public resource, which may or may not be appropriate, have sufficient resources, have a suitable meta-data schema, include compatible ontologies or accommodate adequate data provenance. Data sharing is variably successful and the challenges of Big Data makes this lofty goal far more difficult than it already is.

A robust and reliable infrastructure is a necessity for supporting Big Data sharing intended to serve a global scientific community. Given the potential costs in accommodating Big Data, judicious allocation of resources is needed to insure the widest possible access. The National Institutes of Health recently released an RFA called Big Data to Knowledge (BD2K)(RFA-HG-13-009) whose mission ‘is to enable biomedical scientists to capitalize more fully on the Big Data being generated by those research communities’ (http://bd2k.nih.gov, http://BD2K.org). However, along with the development of more and better technologies to handle Big Data, equally vital is the creation of comprehensive and coherent guidelines, policies and procedures for Big Data access, collaboration and sharing. These policies need to ensure data security, appropriate levels of administrative checks and balances, community governance, as well as promote the creation, maintenance, and support of broad stakeholder trust. Policies necessary to achieve widespread, fair and consistent adoption and to maximize data utility amplify the challenges of Big Data sharing.

### Exemplary Big Data archives

It has already been shown that both technological and policy-related factors contribute to efficacious data sharing [23, 24]. Albeit there are many diverse types of open-access biomedical data archives, we illustrate several examples of popular services that support open collaborative science to maximize the value of their respective data, infrastructure and resources. The Database of Genotypes and Phenotypes (dbGap, http://www.ncbi.nlm.nih.gov/gap) is a framework for sharing large datasets obtained by federally-funded projects. dbGaP is supported by the National Institutes of Health as a free repository for archival, curation and distribution of Big Data, which is organized as a hierarchical structure and includes the accessioned objects, phenotypes (as variables and datasets), various molecular assay data (SNP and Expression Array data, Sequence and Epigenomic marks), analyses and other meta-data [25].

**Fig. 2:**
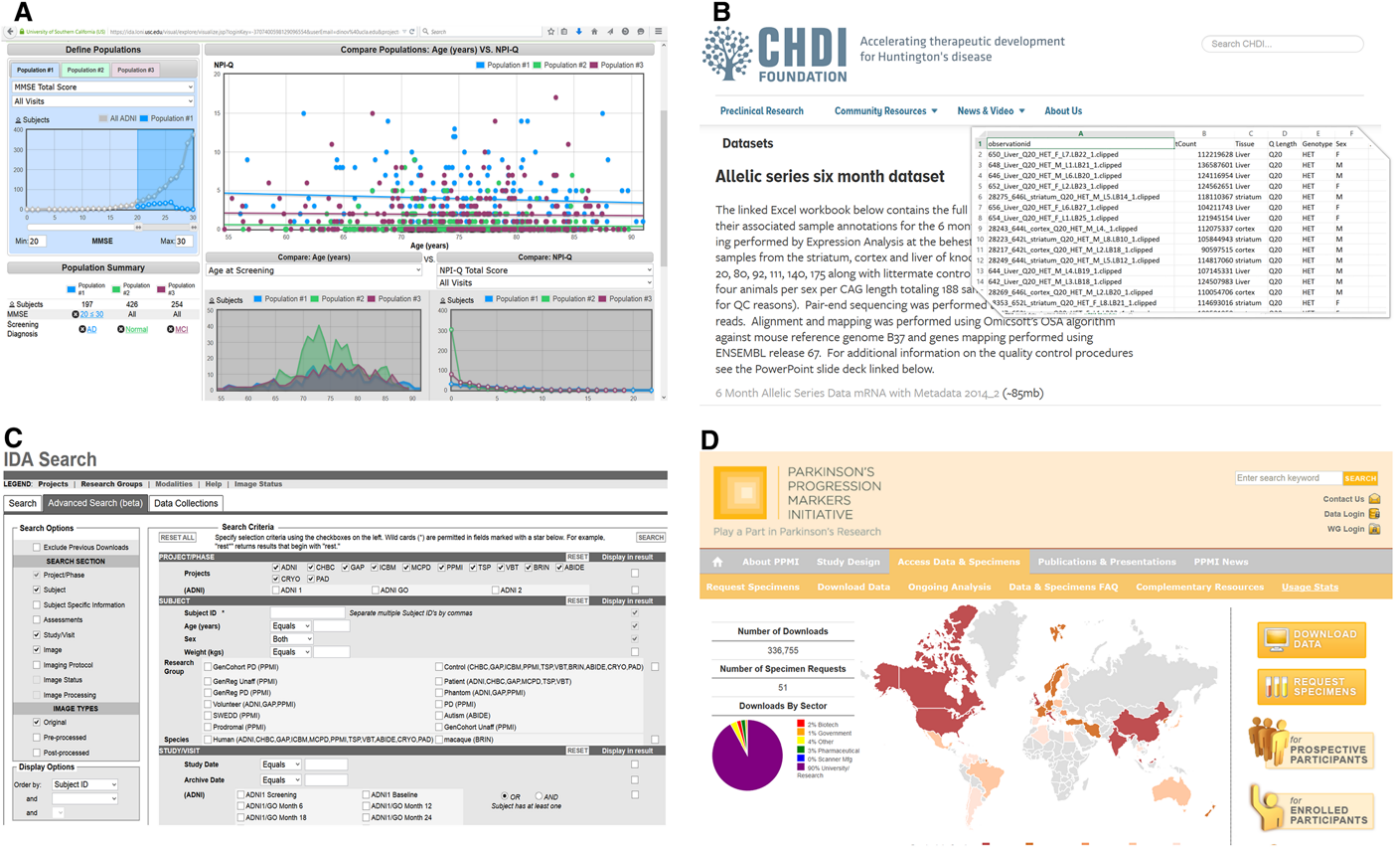
Examples of established Big Biomedical Data archives and analytical platforms

We are already treading the waters of Big Data in our own informatics work on the Alzheimer’s Disease Neuroimaging Initiative (http://adni.loni.usc.edu) [3, 15], Parkinson’s Progression Markers Initiative (http://www.ppmi-info.org) [5], CHDI Foundation (http://chdifoundation.org) [26], the generic imaging-genetics data archive [27, 28], and the Global Alzheimer’s Association Interactive Network (GAAIN) (www.gaain.org) [29], Fig. 2. In these projects, we have encountered the following policy-related factors:

- whether the infrastructure contains viable data and provides flexible methods for data description and relationships among various metadata characteristics (e.g., provenance);
- whether the database is well organized, algorithmically agile and the user access interface is easy to navigate;
- whether the data are derived versions of raw data or the raw data themselves, with the attendant human subjects privacy issues of “extended” consent for Big Data cohort compilation;
- whether the duties and responsibilities of stakeholders, individuals and institutions, are clearly and precisely specified;
- whether clear curation systems governing quality control, data validation, authentication, and authorization are in place;
- whether secure data transactions are efficient and support subsequent data derivation (generation of derived data);
- whether there are pathways and penalties to ensure that requesting investigators give proper attribution to the original and multiple collectors of the data; and
- whether and how the database addresses sociologic and bureaucratic issues germane to data sharing, both open and restricted or tiered access.

As this compilation of factors affecting the day-to-day operations of large-scale data management, processing, and transferring may enable or, if poorly developed or executed, impede scientific discovery, there is an ever-present demand for integrated technological and policy solutions to Big Biomedical Data sharing.

## Findings

### Existent platforms for sharing biomedical data

There is a wide spectrum of architectures currently used for managing and disseminating large-scale health and biomedical datasets. The Cancer Imaging Archive (TCIA) is a component of the Quantitative Imaging Network (QIN) designed to support high-throughput research and development of quantitative imaging methods and candidate biomarkers for the measurement of tumor response in clinical trial settings [30]. TCIA-QIN facilitates data sharing of multi-site and complex clinical data and imaging collections. The Cancer Translational Research Information Platform (caTRIP) [31] promotes data aggregation and query across caGrid data services, joining common data elements, and meta-data navigation (https://github.com/NCIP/catrip). The cBio Cancer Genomics Portal (http://CBioPortal.org) is another open-access resource enabling interactive exploration of multidimensional data sets [32]. The integrating data for analysis, anonymization, and sharing (iDASH) is a cloud-based platform for development and sharing of algorithms and tools for secure HIPAA-compliant data sharing [33]. tranSMART allows novice, intermediate and expert users to collaborate globally, utilize the best analytical tools, establish and communicate convergent standards, and promote new informatics-enabled translational science in the pharmaceutical, academic, and not-for-profit sectors [34]. The Global Alzheimer’s disease Interactive Network (GAAIN) has created a federated approach linking data from hundreds of thousands of subjects participating in research protocols from around the world. Cohort discoveral and visual data exploration are part of this effort [29]. A recent review contrasting some of the pros and cons of existent data sharing platforms concluded that such systems have to be viewed according to the source funding demands, information content, privacy regulations, requirements for analytical and statistical processing, interoperability and scalability needs [35].

### Big Data policy framework

Any set of recommendations for sharing Big Data would depend on the application domain, local, state and federal guidelines, and feedback from all constituents, including funding agencies and the broader research community. Below we outline several categories that might help structure discussions largely based upon our previous experience in our own medium and Big Data informatics cores [14, 21, 36, 37]. These are mostly drafted from the domain of computational neuroimaging and genetics from federally funded investigators and projects but should apply generally to other domains.

### Policies for storing and securing data and ensuring human subjects protection

The importance of protecting the interests of human study participants is paramount and every effort must be made to safeguard subject confidentiality. Any framework for discussing sharing of Big Data must include steps to protect human subject data. That said, HIPAA (the Health Insurance Portability and Accountability Act of 1996) and the sometimes idiosyncratic interpretation of those rules by investigators and local IRBs (Institutional Review Boards) has been at the core of more misinformation, misinterpretation and obfuscating excuse making than any other well intentioned law. Fault lies everywhere. The original intent of HIPAA was (partly) to improve electronic communication of health records and required strict rules to ensure privacy given the ease with which such information could be distributed. Anonymized and de-identified data each have less restriction than patient or subject identifying data. It is far simpler (assuming the science can be conducted) to find a way to conduct the research with anonymized or de-identified data and it is straightforward to remove or replace (as defined in the HIPAA Limited Data Set definition) all subject identifiers prior to the data being stored. If there is a need to retain PHI (Patient Health Information) in the data, broad and or distributed usage is extremely difficult. This may require ‘honest broker’ mechanisms to insulate access to sensitive identifying data only to those properly authorized and authenticated [38, 39]. It is beyond the scope of this article to cover all the security nuances associated with each data type but there are several extra challenges associated with Big Data when data resources must be utilized that are beyond direct control such as distributed or cloud based services. Examples of specific Big Data security challenges include collection, processing, de-identification and extraction of computationally tractable (structured) data. Data aggregation, fusion, and mashing are common practice in Big Data Analytics, however this centralization of data makes it vulnerable to attacks, which can be frequently avoided by properly controlled, protected and frequently inspected (e.g., data-use tracking) access.

Solutions to some of these Big Data managing problems may involve information classification, on-the-fly encoding/decoding of information, implementation of information retention periods, sifting, compression of scrambling meta-data with little value or time-sensitive data that can be disposed in due course, and mining large swathes of data for security events (e.g., malware, phishing, account compromising, etc.) [40]. Finally, Big Data access controls should be managed closer to the actual data, rather than at the edge of the infrastructure, and should be set using the principle of least privilege. Continuously monitoring, tracking and reporting on data usage may quickly identify security weaknesses and ensure that rights and privileges are not abused. Security Information and Event Management (SIEM) and Network Analysis and Visibility (NAV) technologies and data encoding protocols (encryption, tokenization, masking, etc.) may be used to log information from applications, network activity and service performance and provide capabilities to capture, analyze and flag potential attacks and malicious use or abuse of data access [41, 42].

Because cloud based services are distributed and remote, not only are regulatory compliance issues potentially more complicated, but so are monitoring, logging and supporting. The need to know who has touched what data and when they did so are often requirements of legal regulations or funder reporting obligations. Furthermore, project constraints may demand detailed accounting of data utilization. Certainly, monitoring, logging and accounting are of interest to anyone interested in the cost-benefit ratios associated with sharing Big Data. All (especially Cloud based) data storage should require password authentication for any access and all should be logged [43]. For some Big Data which cannot be completely and reliably de-identified [44] or have been censored [45], certain clearance by institutional vetting and specialized secure data access may be justified.

### Policies and processes for data sharing

There are many models of data sharing. Some are fully open, BSD (Berkeley Software Distribution) [46] style (a family of permissive free software licenses, imposing minimal restrictions on the redistribution of covered software) with no attachments or control associated with them. In the realm of Big Data, these are rare and often with limited value because the data may be incomplete, poorly described, improperly collected, outdated or heavily redacted. Obtaining data from other than the acquirer of that data affords the opportunity for it to become corrupted, eroded or tainted along the way, without attribution as its pedigree is undocumented. At the other end of the spectrum, data sharing is barely allowed, with such draconian requirements and specifications that sharing is effectively impeded. These requirements may include rules about scientific purposes for the request, authorship inclusion, limiting access until all project participants publish papers first, and other restrictions. More often are the purported philosophies to share data but without clear requirements or procedures and attempts to actually gain access to the data are met with successive clarification requests, additional prerequisites and delays until the requester gives up all hope and quits.

The fundamental policies for managing Big Data need to specifically address data access, data use and governance, data provenance and distribution, data efficiency, data sharing and result reproducibility. Below we make some concrete recommendations for each.

### Accessibility

Successful models of data sharing usually subscribe to several common themes. 1) They protect data from unauthorized access and ensure equitable access to and distribution of data, without preferential consideration of requests. Because shared databases often contain data owned by both the archivists and collaborating investigators, special privileges by distinct classes of users should be avoided but if required should be explicitly legislated and declared.

Ownership of the data has legal and practical connotations. For the purposes of data sharing policies, owners may be the acquirers of the data, or project leaders or even funders. In the United States, sole ownership or exclusive rights to primary data can be declared legal by the University or institution at which the investigator is employed. Justification can be either ownership of intellectual property or to enable future examination for compliance with regulatory requirements. This was cemented as a result of the Bayh–Dole Act or Patent and Trademark Law Amendments Act of 1980. Institutions can interpret this ruling when irritated by departing faculty and attempt to lay claim to even digital (infinitely replicable) data with limited or no commercial value. Practices such as this get murkier (and nastier) given that shared databases may contain data from collaborating investigators (at other institutions), and/or have explicit data use agreements in place where the host institutions may not have any rights. Even though institutional claims of exclusive ownership are rare, given that the overarching intent of shared databases is to provide access to wider scientific communities, written and legally binding data openness assurances from the host institution should be considered.

### Data use agreements

The purpose of a data use agreement is to; at least, declare the rules of engagement and to describe what is expected of the user, and to some degree, of the provider. Usually it includes explicit human subject protection clauses, authorship expectations, reporting requirements and other guidelines regarding how the data can be used. Often they are annual agreements, requiring an update or re-application each year. Annual expirations are prudent in terms of security, logging accuracy and accounting.

If the owner of the data is considered the acquirer and data depositor, the data use agreement should include expectations and requirements from them as well. Perhaps the most difficult aspect of aggregating data from multiple sources, aside from the methodological variation in its creation, is the variation in degree of description and terms used to describe the data. Data use agreements can be used to declare a minimum standard for upload and inclusion.

Data use agreements can be used to assess the qualifications of both data depositor and data user. Metrics such as quality of data against standardized metrics such as phantoms, for example or other quantitative measures can qualify depositors. Users of the data may also need to be qualified especially if there are real costs associated with delivering the data. Can the user accommodate the volume of data? Have they already requested the same data in the recent past? Are they adhering to the rules of the data use agreement in the past, such providing usage updates, crediting the data source or observing authorship rules?

Data Use Agreements should consider the following;

1. List the permitted uses and disclosures of the data
2. Establish who is permitted to use or receive the data
3. Establish rules and requirements for acknowledgement of the data source, crediting of the project, funder and others as required.
4. Ensure that the recipient or investigator will:
  - Not use or further disclose the information other than as permitted in the agreement or as required by law;
  - Use appropriate safeguards to prevent use or disclosure of the information other than as provided in the agreement;
  - Report to the archive administrators any unpermitted uses or disclosures;
  - Ensure that anyone to whom s/he provides the data (if allowable) agrees to the same restrictions and conditions with respect to the information;
  - Not attempt to identify the information or contact the individuals from whom the data was collected.
5. Agree to provide study results at the conclusion of their investigations (if required).
6. Investigators depositing data may need to:
  - Possess a valid IRB approval or Certification of Exemption from IRB Review for prospective studies [47].
  - Provide a copy of their Standard Operating Procedures document.

In order to effect whatever rules are established and to insure that any (if there are any) applicant qualifications are met, some type of application process may be warranted. Without placing undue burden on the applicant, descriptions of specific research hypotheses and rationale for why the requested data set is suitable along with analytic plan, might be informative.

Perception of fairness and openness are important. Therefore, an independent access control administrator (not the archivist else there may be the perception of too much control concentrated by one entity) should review the request, evaluating the credentials of the requestor and the scientific merit of the proposed project as stipulated in the data use policy. In most cases dealing with human subject data, the requestor will provide all relevant information including: i). Copy of approved IRB or Certification of Exemption from IRB Review (if applicable), ii). Completed and signed of Data Use Agreement or Data Deposition Agreement, iii). Copy of Standard Operating Procedures document (if applicable).

A data archive system can automatically log all data accesses, providing an audit trail for each subject’s data. Finally some form of communication with the data user to obtain a copy of the study results is advisable.

### Data value

Sharing data that is incomplete, incompletely described, of poor or antiquated resolution or quality has little value. It can negatively impact future science because effort is expended re-using data that can either mislead or discourage further examination of hypotheses. Comprehensive provenance and ancillary materials greatly extend the utility of the shared data. These ancillary materials might be full descriptions of the overarching objectives and specific aims of the initial data collections along with descriptions of the kinds of data sets acquired (or in process of being acquired), and instruction on how to utilize aspects of the project infrastructure for other relevant areas of research. Education and training materials covering the spectrum of Big Data acquisition, management, processing, analysis, visualization, protocols and best practices may offer critical means by which to extend the overall reach and value of the information contained in the data [48, 49].

### Policies for achieving cost efficiencies in Big Data sharing

Delivering Big Data often requires more than one solution. Requesters of the data may be able only to accommodate certain technologies. For this reason it is wise to provide multiple technologic solutions to minimize limits and accentuate advantages: FTP (file transfer protocol), GridFTP and other transfer protocols [50, 51], distributed/replicated web-services [52], multiple mirror sites nationwide (a federated model), data compression [53], etc. Other efficiencies can be achieved by organizing and packaging the data for download, such as by subject or genome regions, so that requesters have options. The capability for subsampling the data and perusal of metadata prior to download reduces unnecessary downloads and strain on the infrastructure. Also, sharing resources so that data can be queried, accessed or partially processed via distributed computing pipeline services and retaining pre-processed and processed data for re-use and repurposing is cost effective.

### Cloud based Big Data

Much has been said about cloud based solutions for Big Data [54, 55]. Given available network speeds, most proponents of cloud based solutions argue that proximity between the data store and the compute resources is necessary [56, 57]. Software as a Service (SaaS) [58, 59], representing any software application or a webapp accessible through the Cloud, and Platform as a Service (PaaS) [60], cloud-based service for engineers to create or customize software applications, represent the core of contemporary Cloud Services. Cloud computing functions such as data storage and processing typically require the development of Infrastructure as a Service (IaaS) [61] that ties SaaS and PaaS. Examples of powerful Big Data Cloud services include Google Cloud Platform (https://cloud.google.com/products), Amazon Cloud Services (http://aws.amazon.com), IBM Cloud Services www.ibm.com/cloud, which facilitate secure data access, migration, storage, retrieval, and computational processing [62]. The critical problems with many of these services include the barriers involved in transferring large amounts of data (terabytes) and the lack of efficient mechanisms for agile and efficient deployment and management of innovative analytics platforms, including open-source machine learning, data wrangling, classification and visualization tools [63–65].

### Sharing sociology

Big Data sharing in the biomedical sciences can present sociological challenges. Researchers can be wary of open-sharing initiatives and thus may be reluctant to provide their data if they view data contribution as a one-way street. Data sharing in the neurosciences provides a valuable example. When scientists have a say in data access and are ensured appropriate attribution, these concerns can be mitigated. Big Data initiatives are therefore ideally predicated on a stakeholder model in which policies for sharing will be enhanced and publicized with reports on the number of views, downloads and derived data processing, and when their data is being accessed and by whom, among other benefits and services. In this manner, original data contributors are active participants in the value added that sharing produces. Likewise, these contributing scientists will feel confident that they will receive all appropriate attribution afforded to them in the use of their data by others. To help the participants of a given study or trial appreciate the volume of sharing, database investigators and staff must work closely with the users to realize the potential benefits to be gained for data that are shared as openly as possible.

With care and thoughtfulness, Big Data sharing can be realized to the benefit of all and ensure that each data initiative serves as an important and honest broker for the openness of health sciences information important to the scientific community at large as well as targeted patient populations and advocates.
